# Nomogram Predicts Risk and Prognostic Factors for Bone Metastasis of Pancreatic Cancer: A Population-Based Analysis

**DOI:** 10.3389/fendo.2021.752176

**Published:** 2022-03-09

**Authors:** Wei Zhang, Lichen Ji, Xijun Wang, Senbo Zhu, Junchao Luo, Yin Zhang, Yu Tong, Fabo Feng, Yao Kang, Qing Bi

**Affiliations:** ^1^ Department of Orthopedics, Zhejiang Provincial People's Hospital, Qingdao University, Qingdao, China; ^2^ Department of Orthopedics, Zhejiang Provincial People’s Hospital, Hangzhou, China; ^3^ Department of Orthopedics, The Second Affiliated Hospital of Wenzhou Medical University, Wenzhou, China; ^4^ Department of Head and Neck Surgery, Sun Yat-sen University Cancer Center, Guangzhou, China; ^5^ State Key Laboratory of Oncology in South China, Sun Yat-sen University, Guangzhou, China; ^6^ Collaborative Innovation Center for Cancer Medicine, Sun Yat-sen University, Guangzhou, China; ^7^ Graduate Department, Bengbu Medical College, Bengbu, China; ^8^ Department of Orthopedics, Hangzhou Medical College People's Hospital, Hangzhou, China

**Keywords:** pancreatic cancer, bone metastasis, predictors, Surveillance Epidemiology and End Results (SEER) database, logistic regression, Cox regression, nomogram

## Abstract

**Background:**

The overall survival (OS) of pancreatic cancer (PC) patients with bone metastasis (BM) is extremely low, and it is pretty hard to treat bone metastasis. However, there are currently no effective nomograms to predict the diagnosis and prognosis of pancreatic cancer with bone metastasis (PCBM). Therefore, it is of great significance to establish effective predictive models to guide clinical practice.

**Methods:**

We screened patients from Surveillance Epidemiology and End Results (SEER) database between 2010 and 2016. The independent risk factors of PCBM were identified from univariable and multivariable logistic regression analyses, and univariate and multivariate Cox proportional hazards regression analyses were used to determine independent prognostic factors affecting the prognosis of PCBM. In addition, two nomograms were constructed to predict the risk and prognosis of PCBM. We used the area under the curve (AUC), C-index and calibration curve to determine the predictive accuracy and discriminability of nomograms. The decision curve analysis (DCA) and Kaplan-Meier(K-M) survival curves were employed to further confirm the clinical effectiveness of the nomogram.

**Results:**

Multivariable logistic regression analyses revealed that risk factors of PCBM included age, primary site, histological subtype, N stage, radiotherapy, surgery, brain metastasis, lung metastasis, and liver metastasis. Using Cox regression analyses, we found that independent prognostic factors of PCBM were age, race, grade, histological subtype, surgery, chemotherapy, and lung metastasis. We utilized nomograms to visually express data analysis results. The C-index of training cohort was 0.795 (95%CI: 0.758-0.832), whereas that of internal validation cohort was 0.800 (95%CI: 0.739-0.862), and the external validation cohort was 0.787 (95%CI: 0.746-0.828). Based on AUC of receiver operating characteristic (ROC) analysis, calibration plots, and decision curve analysis (DCA), we concluded that the risk and prognosis model of PCBM exhibits excellent performance.

**Conclusion:**

Nomogram is sufficiently accurate to predict the risk and prognostic factors of PCBM, allowing for individualized clinical decisions for future clinical work.

## Introduction

According to 2020 cancer statistics report, pancreatic cancer (PC) is the seventh leading cause of cancer death in both sexes, accounting for numerous deaths due to its poor prognosis. The incidence rate of PC is higher in countries with a higher human development index, and its incidence has remained stable over time (1). According to a study, PC is predicted to overtake breast cancer as the third leading cause of cancer death in 28 European countries by 2025 (2). This indicates that PC exhibits a high incidence and mortality in digestive system tumors. Despite significant advances in detecting and treating PC, only 4% of patients survive five years after diagnosis (3).

Cancer metastasis involves a multi-step invasion and metastasis cascade process. Under complex gene regulation mechanisms, primary tumor cells migrate away from the primary site to other sites and gradually grow into secondary tumors (4, 5). We are concerned about cancer metastasis because it is responsible for 90% of cancer deaths, not the primary tumor (6). Bone is the third most common site of metastasis for solid tumors (7). In addition, complications such as pain, pathological fracture, nerve root or spinal cord compression, hypercalcemia, and severe bone marrow infiltration caused by bone metastasis significantly affect patients’ quality of life (8). Therefore, the survival rate of pancreatic cancer with bone metastasis (PCBM) patients has remained low.

The different incidence of PCBM reported in literature (from 5% to 20%) should depend on either the possible overlapping between bone localization and symptoms associated with the primary tumor or the longer survival obtained in the past few years due to new and more effective chemotherapy regimens in both adjuvant and advanced settings (9–12). There seems to be some suggestion that patients who have a primary that is in the tail of the pancreas are more likely to develop bone metastasis (13). Bone surveys using standard roentgenograms, CT scans, MRIs, and positron emission topographic (PET) scans have been used to detect skeletal metastases in pancreatic cancer (14–17). It seems that no imaging modality appears to have a superior detection rate. However, when used in conjunction, the rates of detection may be much higher. In terms of treatment, the literature shows that a first-line chemotherapy regimen was administered with gemcitabine plus nab-paclitaxel in combination with zoledronic acid (18). The previous clinical reports on PCBM primarily focused on case reports and single-institutional cohort studies (19–21). Due to the small sample size and low credibility of these studies, there is an apparent deficiency in their guiding value for clinical practice. In addition, due to the relatively low incidence of PCBM, most current treatment schemes for multi-directional control of PC from clinical experience. Therefore, we are in urgent need of practical tools and concise guidelines for clinical treatment.

Nomograms are widely used in cancer prognosis and recurrence mainly because they can simplify statistical prediction models to a single numerical estimate of the probability of events (such as death or recurrence) depending on the situation of individual patients (22–24). A user-friendly graphical interface can generate insights in the clinical process, thus promoting nomogram use for clinical decision-making (25). For many cancers, nomograms are superior to traditional TNM staging systems (26) and have become a new standard (27, 28). As far as we know, no model has been developed to predict the overall survival (OS) of PC patients with BM. As a result, we want to construct and validate nomogram, and use it to predict 1-, 2-, 3-year OS in PCBM.

## Materials and Methods

### Patient Selection

Data on newly diagnosed PC patients from 2010 to 2016 were extracted from SEER database, which was the largest cancer database in the United States, containing information on survival characteristics and incidence of malignant tumors in 26% of the population of 18 cancer registries in the country (29). The patients we collected must meet the following criteria: (1) patients must have complete data regarding their survival time; (2) the effectiveness of follow-up must be ensured; (3) the source of the case must remove all cases obtained through autopsy and retain only those identified on the death report. (4) Pancreatic cancer was diagnosed by pathology, and bone metastasis could be diagnosed by imaging. Finally, 19067 patients diagnosed with pancreatic cancer were included in the present study, including 235 patients who had BM. Besides, we retrospectively collected the data of PC patients with BM in Zhejiang Provincial People’s Hospital and Sun Yat-sen University Cancer Center between 2010 and 2020 as an external validation cohort for our study. The inclusion and exclusion criteria of external validation cohort were consistent with those of internal cohort. Informed consent was obtained from all patients before patient inclusion, and this study was approved by the Ethics Committee of Zhejiang Provincial People’s Hospital.

### Data Elements

We collected data on the following baseline characteristics of PC patients: age at diagnosis, race, sex, histological subtype, grade, primary site, TNM stage, surgery, radiotherapy, chemotherapy, tumor size, brain metastasis, liver metastasis, and lung metastasis. In addition, histological codes were divided into four categories mainly based on the International Classification of Diseases for Oncology (ICD-O): adenocarcinoma (histologic codes 8140,8480), infiltrating duct carcinoma (histologic code 8500), neuroendocrine carcinoma (histologic code 8246), and others (histologic codes 8010, 8012, 8013, 8020, 8021, 8041, 8046, 8070, 8150, 8240, 8244, 8249, 8481, 8490, 8560). The best tumor size cut-off value of OS were determined by x-tiles software (30). The subjects were divided into three groups according their tumor size: large, medium, and small groups. In survival analysis, the main end point of our study was OS, which was defined as the date from diagnosis to death (for any reason) or the date of the last follow-up. For the external validation cohort, we used the electronic medical record system to collect baseline characteristics of patients with pancreatic cancer. Because of the ethnic differences between China and the United States, we did not include race in our study. In survival analysis, we recorded patient’s OS by phone follow-up.

### Statistical Analysis

We used R software (version 4.0.5) to analyze the data in this research. For statistical methods, the independent t-test or Mann-Whitney U test were utilized to compare continuous data, while the chi-square test or Fisher exact test were deployed to compare categorical data. All variables were subjected to univariable logistic analysis, and those with a P <0.05 were incorporated into multivariable logistic analysis to determine the risk factors for BM in PC patients. Then, we analyzed the survival of 235 PCBM patients to ascertain its prognostic factors. All patients were randomly divided into training (n=167) and internal validation (n=68) cohorts according to the proportion of 7:3. We performed univariate Cox proportional hazard regression analysis on all variables and included those with a P <0.05 into multivariate Cox proportional hazard regression analysis to determine independent prognostic factors of PCBM. In addition, we established two nomograms based on risk factors and independent prognostic factors to predict the risk and OS of PCBM. The accuracy of nomograms was evaluated using C-index and ROC, and the discrimination of nomograms was verified using calibration plots. DCA is a method to evaluate the clinical utility of different predictive models (31). It can compare the difference between nomogram and other models by quantifying the net income under different threshold probabilities. Since DCA can display the false- and the true-positive fractions as functions of the risk threshold, it compensates for any deficiency of ROC curves (32). In this study, P <0.05 (bilateral) was considered statistically significant.

## Results

### Patients Baseline Clinical Characteristics

According to our rigorous screening, our study included 19067 PC patients from SEER database. Among them, 235 patients had PCBM, 167 cases served as training cohort, and the remaining 68 patients were internal validation cohort. **Table 1** presents baseline clinical features and treatment regimens of pancreatic carcinoma patients. Significant differences were detected between PC without BM and PC with BM in age (Median:67y Range:59-75y vs Median:65y Range:56-73y, P<0.01). It was found that whites accounted for 80.12% and the most common histological subtype was adenocarcinoma (51.50%). Grade II (41.14%) was the most common degree of differentiation. Among primary sites, PC was most likely to occur in the head of pancreas (58.56%). In addition, the most common stages of T and N were T3 (57.77%) and N1 (51.51%). In terms of treatment, 12309 cases (64.56%) underwent surgery, 4144 cases (21.73%) underwent radiotherapy, and 10997 cases (57.68%) underwent chemotherapy. Regarding tumor size, 4-38mm accounted for 59.20%. In distant metastases of pancreatic cancer, there were 21 cases of brain metastasis (0.11%), 3224 cases of liver metastasis (16.91%), and 805 cases of lung metastasis (4.22%).

**Table 1:** Baseline clinical features and treatment regimen of pancreatic carcinoma patients.

Characteristics	Without BM number (n=18832)	With BM number (n=235)	χ^2^	*P*
Age				0.005^*^
Median	67	65		
Range	59-75	56-73		
Race			0.074	0.964
White	15087	189		
Black	2103	25		
Other	1642	21		
Sex			4.525	0.033
Female	9087	97		
Male	9745	138		
Histological subtype			43.220	<0.001
Adenocarcinoma	9669	151		
Infiltrating duct carcinoma	5090	21		
Neuroendocrine carcinoma	1405	29		
Other	2668	34		
Grade			44.831	<0.001
Well differentiated: I	4032	27		
Moderately differentiated: II	7757	87		
Poorly differentiated: III	6644	104		
Undifferentiated; anaplastic: IV	399	17		
Primary Site			78.441	<0.001
Head of pancreas	11091	74		
Body of pancreas	2269	36		
Tail of pancreas	3082	71		
Pancreatic duct	82	2		
Other specified parts of pancreas	299	8		
Overlapping lesion of pancreas	1213	26		
Pancreas, NOS	796	18		
AJCC T stage			69.506	<0.001
T1	1762	10		
T2	3653	75		
T3	10926	89		
T4	2491	61		
AJCC N stage			4.385	0.036
N0	9147	98		
N1	9685	137		
Surgery			331.616	<0.001
No	6542	216		
Yes	12290	19		
Radiotherapy			7.256	0.007
No	14756	167		
Yes	4076	68		
Chemotherapy			0.038	0.846
No	7972	98		
Yes	10860	137		
Tumor size			47.949	<0.001
4-38 mm	11196	92		
39-67 mm	6251	106		
68-150 mm	1385	37		
Brain metastasis			371.606	<0.001
No	18821	225		
Yes	11	10		
Liver metastasis			393.402	<0.001
No	15761	82		
Yes	3071	153		
Lung metastasis			553.540	<0.001
No	18109	153		
Yes	723	82		

^*^Mann-Whitney U test.

### Independent Risk Factors for PCBM

As shown in **Table 2**, we conducted the univariable logistic analysis on fifteen potential factors, and the result determined thirteen BM-related variables, including age, sex, histological subtype, grade, primary site, T stage, N stage, surgery, radiotherapy, tumor size, brain metastasis, liver metastasis, and lung metastasis. Additionally, the multivariable logistic regression analysis revealed that independent predictors of PCBM were age, histological subtype, primary site, N stage, surgery, radiotherapy, brain metastasis, liver metastasis, and lung metastasis.

**Table 2:** Univariable and multivariable logistic regression of risk factor of bone metastasis in pancreatic carcinoma patients.

Characteristics	Univariate analysis		Multivariate analysis	
	OR (95%CI)	*P*	OR (95%CI)	*P*
Age	0.984 (0.974-0.995)	0.004	0.979 (0.967-0.990)	<0.001
Race				
White	Reference			
Black	0.949 (0.609-1.415)	0.807		
Other	1.021 (0.630-1.568)	0.929		
Sex				
Female	Reference			
Male	1.327 (1.023-1.727)	0.034		
Histological subtype				
Adenocarcinoma	Reference		Reference	
Infiltrating duct carcinoma	0.264 (0.162-0.408)	<0.001	1.023 (0.607-1.647)	0.928
Neuroendocrine carcinoma	1.322 (0.868-1.943)	0.173	2.004 (1.219-3.198)	0.005
Other	0.816 (0.552-1.171)	0.287	1.089 (0.699-1.652)	0.697
Grade				
Well differentiated: I	Reference			
Moderately differentiated: II	1.675 (1.102-2.631)	<0.001		
Poorly differentiated: III	2.338 (1.552-3.645)	0.020		
Undifferentiated; anaplastic: IV	6.363 (3.376-11.668)	<0.001		
Primary Site				
Head of pancreas	Reference		Reference	
Body of pancreas	2.378 (1.576-3.523)	<0.001	1.418 (0.919-2.151)	0.106
Tail of pancreas	3.453 (2.484-4.795)	<0.001	2.793 (1.927-4.046)	<0.001
Pancreatic duct	3.656 (0.595-11.894)	0.074	4.018 (0.616-14.724)	0.072
Other specified parts of pancreas	4.010 (1.768-7.897)	<0.001	2.657 (1.099-5.619)	0.018
Overlapping lesion of pancreas	3.213 (2.011-4.973)	<0.001	1.764 (1.066-2.835)	0.022
Pancreas, NOS	3.389 (1.956-5.569)	<0.001	1.966 (1.110-3.348)	0.017
AJCC T stage				
T1	Reference			
T2	3.618 (1.958-7.471)	<0.001		
T3	1.435 (0.783-2.948)	0.280		
T4	4.315 (2.309-8.976)	<0.001		
AJCC N stage				
N0	Reference		Reference	
N1	1.320 (1.019-1.718)	0.037	1.703 (1.276-2.280)	<0.001
Surgery				
No	Reference		Reference	
Yes	0.047 (0.028-0.073)	<0.001	0.072 (0.041-0.122)	<0.001
Radiotherapy				
No	Reference		Reference	
Yes	1.474 (1.103-1.949)	0.008	3.783 (2.679-5.310)	<0.001
Chemotherapy				
No	Reference			
Yes	1.026 (0.792-1.335)	0.846		
Tumor size				
4-38 mm	Reference			
39-67 mm	2.064 (1.559-2.737)	<0.001		
68-150 mm	3.251 (2.186-4.738)	<0.001		
Brain metastasis				
No	Reference		Reference	
Yes	76.044 (31.387-182.135)	<0.001	11.901 (4.139-33.936)	<0.001
Liver metastasis				
No	Reference		Reference	
Yes	9.576 (7.326-12.607)	<0.001	3.044 (2.197-4.257)	<0.001
Lung metastasis				
No	Reference		Reference	
Yes	13.424 (10.125-17.680)	<0.001	4.071 (2.962-5.566)	<0.001

### Diagnostic Nomogram Model Establishment and Validation

**Figure 1:**
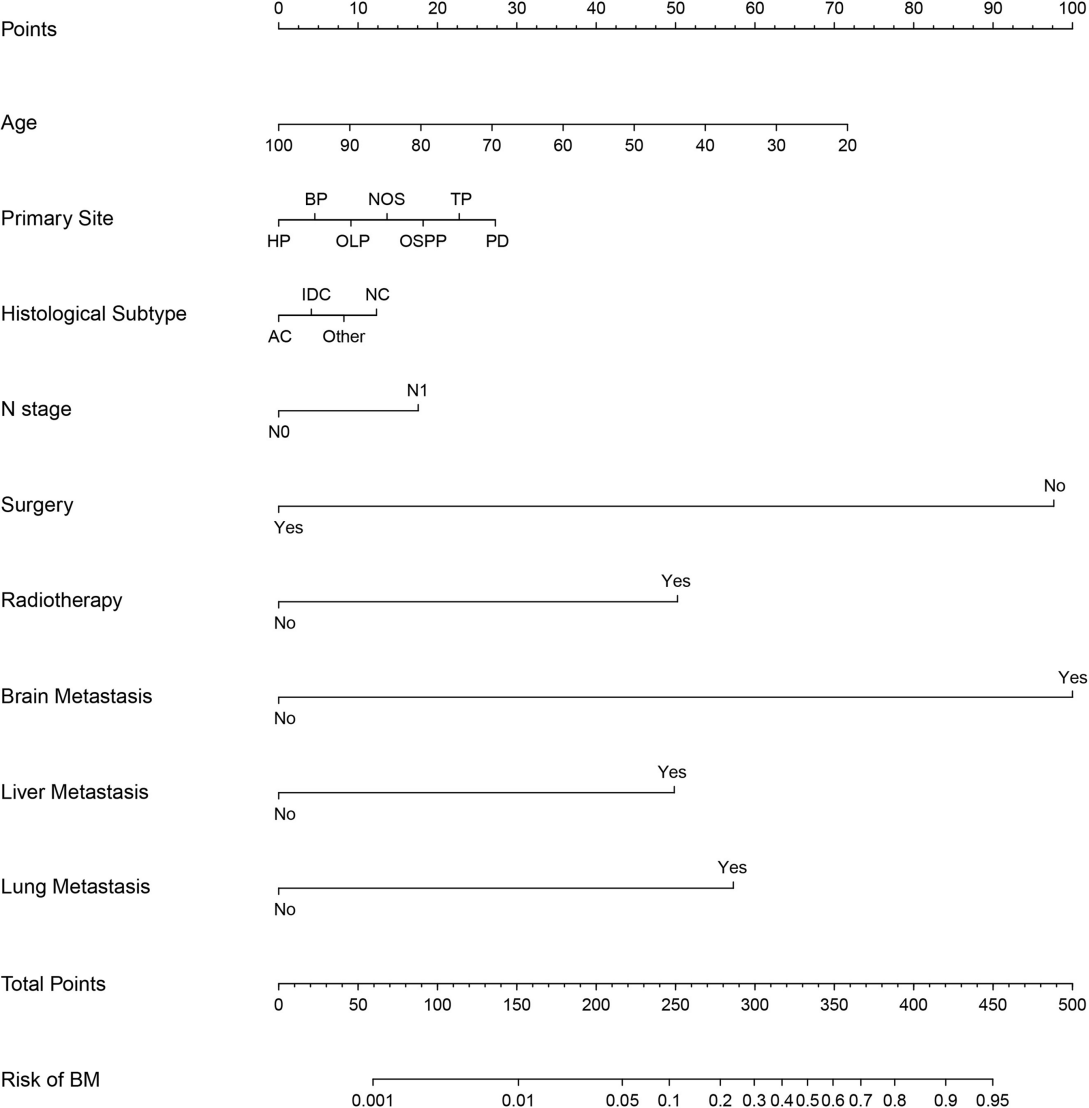
Nomogram to estimate the risk of bone metastasis in patients with pancreatic cancer. HP, head of pancreas; BP, body of pancreas; OLP, overlapping lesion of pancreas; OSPP, other specified parts of pancreas; TP, tail of pancreas; PD, pancreatic duct; AC, Adenocarcinoma; IDC, Infiltrating duct carcinoma; NC, neuroendocrine carcinoma.

**Figure 2:**
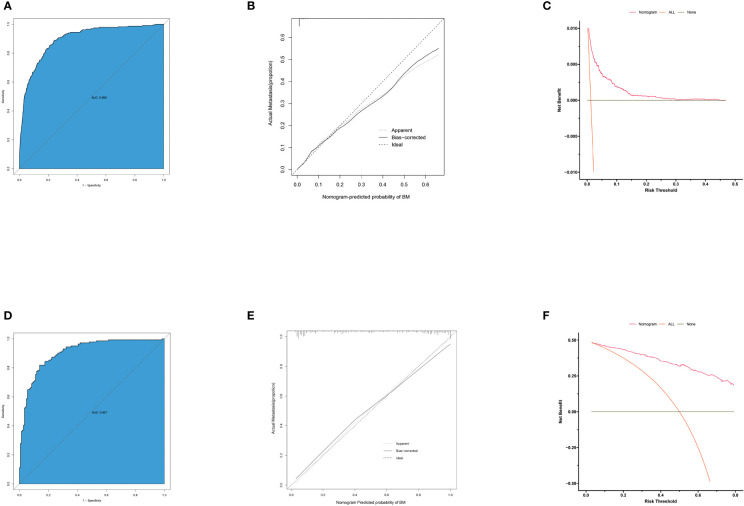
ROC curves, calibration plots and DCA of the nomogram for the risk of pancreatic cancer with bone metastasis. **(A)** The area under ROC curve was utilized to judge the advantages and disadvantages of nomogram. **(B)** Calibration plot for the diagnostic nomogram. The diagonal 45-degree line indicates perfect prediction. **(C)** Decision curve analysis for the diagnostic nomogram. The net benefit calculated by adding true positive and minus the false positive corresponds to the measurement of Y-axis; X-axis represents the threshold probability. **(D)** The area under ROC curve of external validation cohort. **(E)** Calibration plot for diagnostic nomogram in external validation cohort. **(F)** Decision curve analysis for diagnostic nomogram in external validation cohort.

**Figure 3:**
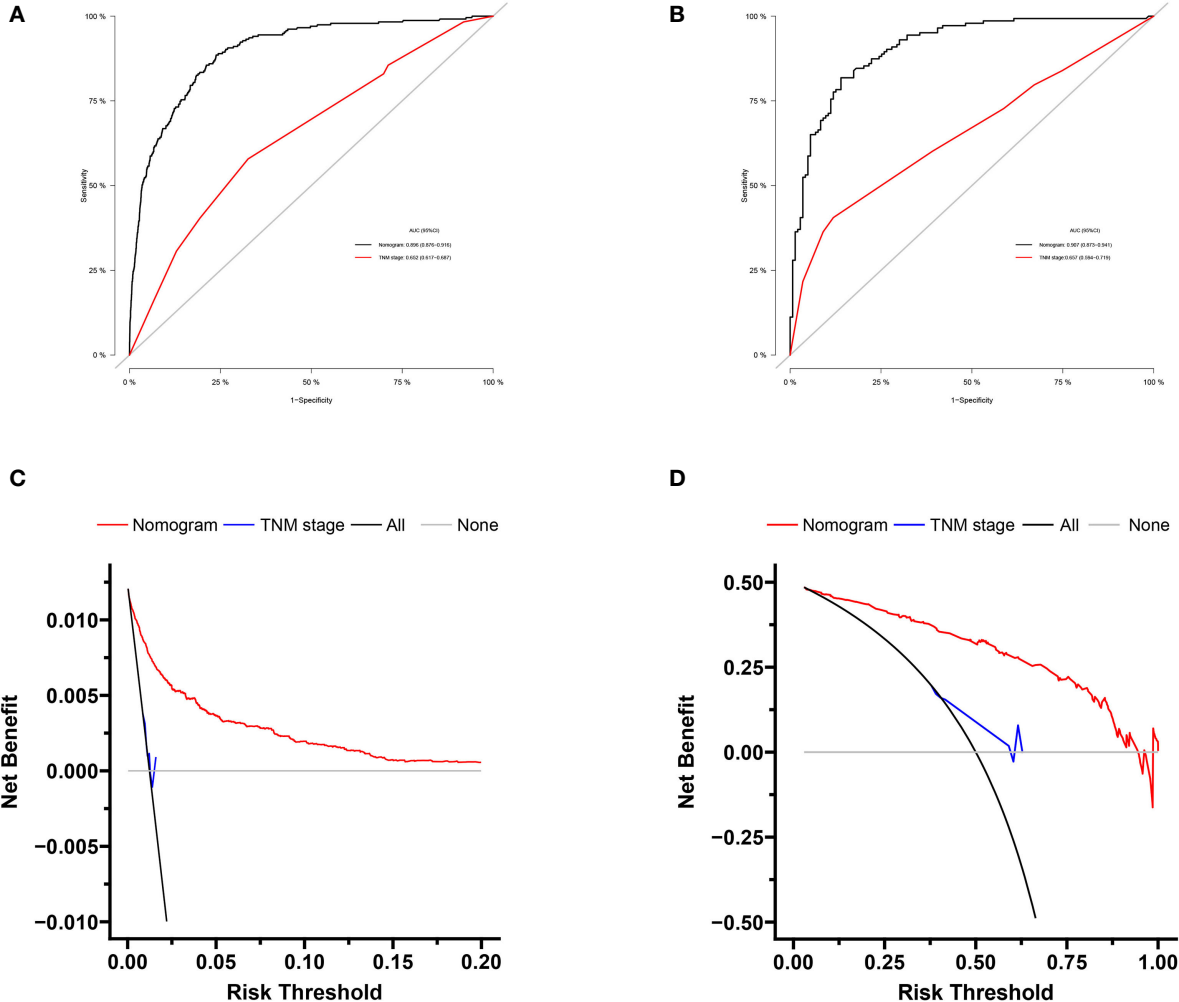
Comparison of area under the receiver operating characteristic curves and DCA curves between nomogram and TNM stage in the training cohort **(A, B)** and external validation cohort **(C, D)**.

Based on independent predictors obtained by multivariable logistic regression, we constructed a risk prediction nomogram model of PCBM (**Figure 1**). In turn, the nomogram was made available *via* a free browser-based online calculator available at https://pcbm.shinyapps.io/DynNomapp/. ROC analysis revealed that AUC value of the nomogram reached 0.896, indicating that this model has excellent discriminant ability (**Figure 2A**). By observing the calibration curve, the observed results were highly consistent with predicted results (**Figure 2B**). In addition, DCA showed that the nomogram model is effective in clinical practice (**Figure 2C**). To further validate the model in the Chinese population, we created an external validation cohort and plotted its corresponding validation curves. ROC analysis revealed that nomogram’s AUC value was 0.907, indicating that this model also has excellent discriminant ability in Chinese population (**Figure 2D**). The calibration curve demonstrated the best consistency between nomogram predictions and actual observations, and the external verification cohort was consistent with the training cohort (**Figure 2E**). In the external validation cohort, DCA also demonstrated that the nomogram model performs well in clinical practice (**Figure 2F**). At the same time, we also plotted ROC and DCA curves of TNM stage, demonstrating a better discriminative ability than TNM stage, both in the training and external validation cohorts (**Figures 3A**–**D**).

### Independent Prognostic Factors for PCBM

**Table 3** displayed information on clinical features and treatment regimens of PC patients with BM. The Chi-square test and Fisher’s exact test indicated that there were no significant differences in all variables between the training cohort and the validation cohort. In the training cohort, we performed a univariate Cox proportional hazards regression analysis. The results demonstrated that age, race, histological subtype, grade, primary site, T stage, surgery, radiotherapy, chemotherapy, and lung metastasis were prognostic factors (P <0.05) (**Table 4**). The variables with P<0.05 were then included in multivariate Cox proportional hazards regression analysis. Finally, it was found that age, race, histological subtype, grade, surgery, chemotherapy and lung metastasis were identified as independent prognostic factors for OS (**Table 4**).

**Table 3:** Demographic and clinicopathological characteristics in pancreatic cancer patients with bone metastasis.

Characteristics	Training cohort (N=167)		Validation cohort (N=68)		χ^2^	*P*
	n	%	n	%		
Age	63.27±12.195		66.41±11.820			0.072^*^
Race					0.220	0.896
White	135	80.84	54	79.41		
Black	18	10.78	7	10.29		
Other	14	8.38	7	10.29		
Sex					2.193	0.139
Female	74	44.31	23	33.82		
Male	93	55.69	45	66.18		
Histological subtype					0.998	0.802
Adenocarcinoma	108	64.67	43	63.24		
Infiltrating duct carcinoma	13	7.78	8	11.76		
Neuroendocrine carcinoma	21	12.57	8	11.76		
Other	25	14.97	9	13.24		
Grade					1.386	0.709
Well differentiated: I	20	11.98	7	10.29		
Moderately differentiated: II	58	34.73	29	42.65		
Poorly differentiated: III	77	46.11	27	39.71		
Undifferentiated; anaplastic: IV	12	7.19	5	7.35		
Primary Site					3.489	0.745
Head of pancreas	49	29.34	25	36.76		
Body of pancreas	28	16.77	8	11.76		
Tail of pancreas	53	31.74	18	26.47		
Pancreatic duct	1	0.60	1	1.47		
Other specified parts of pancreas	6	3.59	2	2.94		
Overlapping lesion of pancreas	19	11.38	7	10.29		
Pancreas, NOS	11	6.59	7	10.29		
AJCC T stage					0.325	0.955
T1	7	4.19	3	4.41		
T2	53	31.74	22	32.35		
T3	65	38.92	24	35.29		
T4	42	25.15	19	27.94		
AJCC N stage					1.129	0.288
N0	66	39.52	32	47.06		
N1	101	60.48	36	52.94		
Surgery					0.625	0.429
No	152	91.02	64	94.11		
Yes	15	8.98	4	5.88		
Radiotherapy					0.283	0.595
No	117	70.06	50	73.53		
Yes	50	29.94	18	26.47		
Chemotherapy					0.157	0.692
No	71	42.51	27	39.71		
Yes	96	57.49	41	60.29		
Tumor size					2.186	0.335
4-38 mm	63	37.72	29	42.65		
39-67 mm	74	44.31	32	47.06		
68-150 mm	30	17.96	7	10.29		
Brain metastasis					0.079	0.779
No	159	95.21	66	97.06		
Yes	8	4.79	2	2.94		
Liver metastasis					0.975	0.323
No	55	32.93	27	39.71		
Yes	112	67.07	41	60.29		
Lung metastasis					0.975	0.323
No	112	67.07	41	60.29		
Yes	55	32.93	27	39.71		

^*^T test.

**Table 4:** Univariate and multivariate Cox proportional hazards regression analysis in pancreatic carcinoma patients with bone metastasis.

Characteristic	Univariate analysis		Multivariate analysis	
	HR (95% CI)	P value	HR (95% CI)	*P* value
Age	1.021 (1.007-1.036)	0.004	1.031 (1.013-1.050)	<0.001
Race				
White	Reference		Reference	
Black	2.186 (1.281-3.732)	0.004	2.208 (1.257-3.877)	0.006
Other	0.806 (0.422-1.540)	0.514	0.718 (0.357-1.443)	0.352
Sex				
Female	Reference			
Male	1.276 (0.909-1.791)	0.158		
Histological subtype				
Adenocarcinoma	Reference		Reference	
Infiltrating duct carcinoma	0.450 (0.234-0.868)	0.017	0.419 (0.209-0.842)	0.015
Neuroendocrine carcinoma	0.237 (0.127-0.444)	<0.001	0.259 (0.134-0.501)	<0.001
Other	0.612 (0.365-1.027)	0.063	0.522 (0.296-0.920)	0.024
Grade				
Well differentiated: I	Reference		Reference	
Moderately differentiated: II	3.810 (1.786-8.131)	<0.001	2.350 (1.097-5.035)	0.028
Poorly differentiated: III	5.194 (2.470-10.925)	<0.001	4.653 (2.191-9.882)	<0.001
Undifferentiated; anaplastic: IV	6.421 (2.502-16.480)	<0.001	6.740 (2.499-18.174)	<0.001
Primary Site				
Head of pancreas	Reference			
Body of pancreas	0.859 (0.516-1.428)	0.557		
Tail of pancreas	0.752 (0.489-1.157)	0.195		
Pancreatic duct	11.786 (1.550-89.624)	0.017		
Other specified parts of pancreas	1.017 (0.401-2.576)	0.972		
Overlapping lesion of pancreas	0.895 (0.509-1.573)	0.699		
Pancreas, NOS	0.966 (0.433-2.154)	0.933		
AJCC T stage				
T1	Reference			
T2	0.530 (0.225-1.251)	0.147		
T3	0.352 (0.150-0.829)	0.017		
T4	0.364 (0.151-0.877)	0.024		
AJCC N stage				
N0	Reference			
N1	1.251 (0.888-1.763)	0.201		
Surgery				
No	Reference		Reference	
Yes	0.416 (0.218-0.795)	0.008	0.323 (0.150-0.695)	0.004
Radiotherapy				
No	Reference			
Yes	0.722 (0.495-1.052)	0.090		
Chemotherapy				
No	Reference		Reference	
Yes	0.638 (0.452-0.900)	0.011	0.286 (0.191-0.430)	<0.001
Tumor size				
4-38 mm	Reference			
39-67 mm	1.243 (0.857-1.802)	0.252		
68-150 mm	1.192 (0.733-1.937)	0.479		
Brain metastasis				
No	Reference			
Yes	1.512 (0.738-3.099)	0.259		
Liver metastasis				
No	Reference			
Yes	1.092 (0.764-1.561)	0.628		
Lung metastasis				
No	Reference		Reference	
Yes	1.575 (1.100-2.254)	0.013	1.978 (1.314-2.976)	0.001

### Prognostic Nomogram Model Establishment and Validation

**Figure 4:**
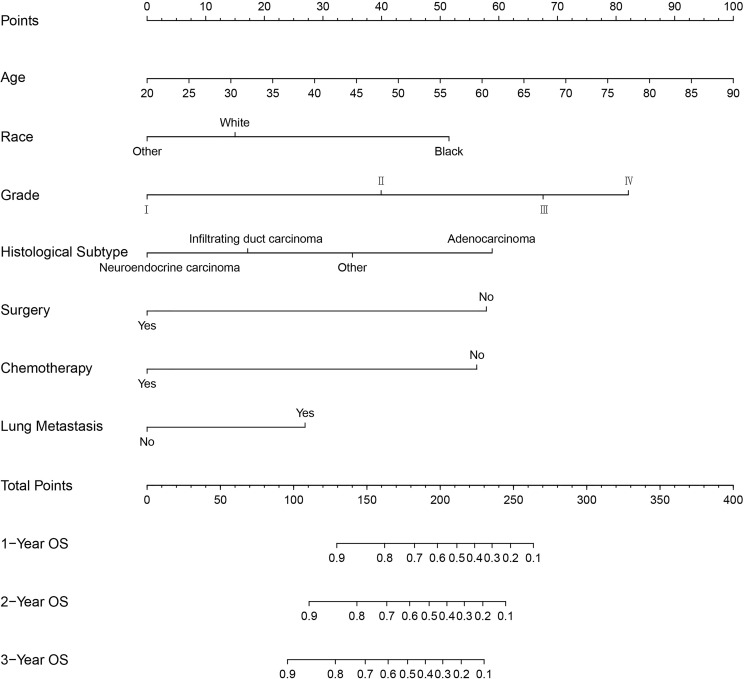
Nomogram for predicting the overall survival of patients with pancreatic cancer presenting with bone metastasis. To use this nomogram, the specific point for each variable of the patient lies on each variable axis. Draw a vertical line upward to determine the point at which each variable accepts; the sum of these points is located on the Total Points axis, and draw a vertical line down to the survival axis to determine the probability of 1-, 2- and 3- year overall survival.

**Figure 5:**
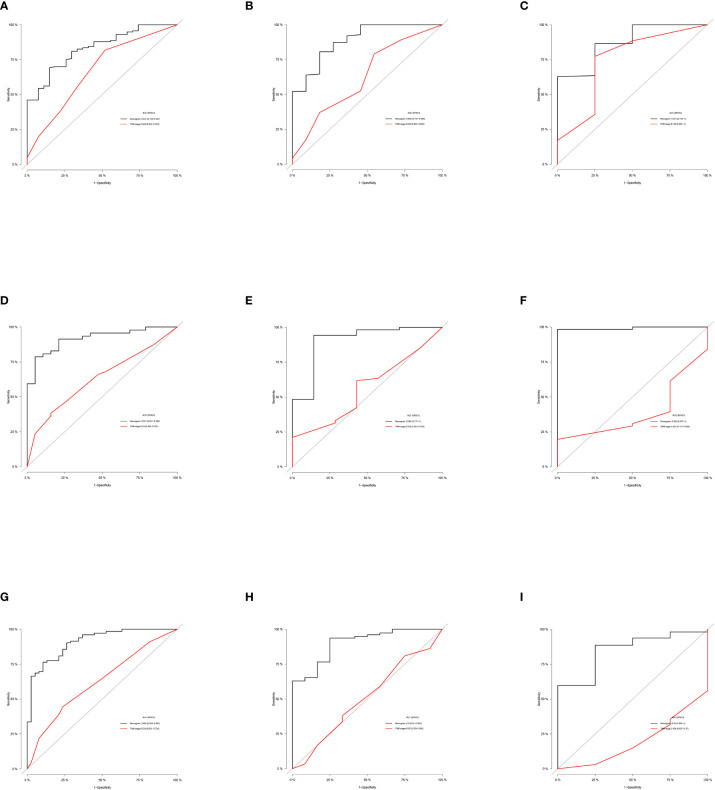
ROC curves of the ability of nomogram and TNM stage to predict 1-, 2- and 3-year overall survival in **(A–C)** training cohort, **(D–F)** internal validation cohort, and **(G–I)** external validation cohort.

**Figure 6:**
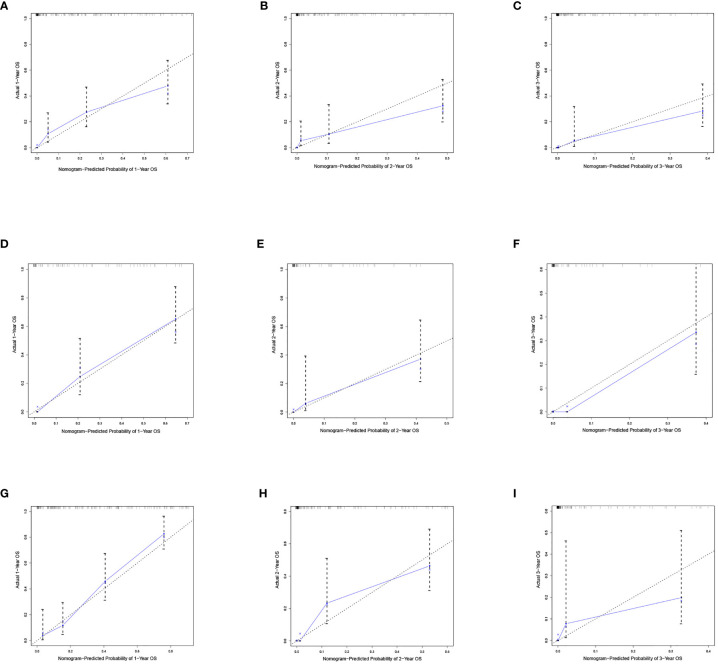
Calibration curves of the nomograms. Calibration curves of 1-, 2- and 3-year overall survival for PCBM patients in **(A–C)** training cohort, **(D–F)** internal validation cohort, and **(G–I)** external validation cohort. The dotted line represents the ideal reference line, where the predicted probability would match the observed survival rate. The blue dots are calculated by bootstrapping (resample:100) and represent the nomogram performance. The closer the solid blue line is to the dotted line, the more accurate the model is in predicting overall survival.

**Figure 7:**
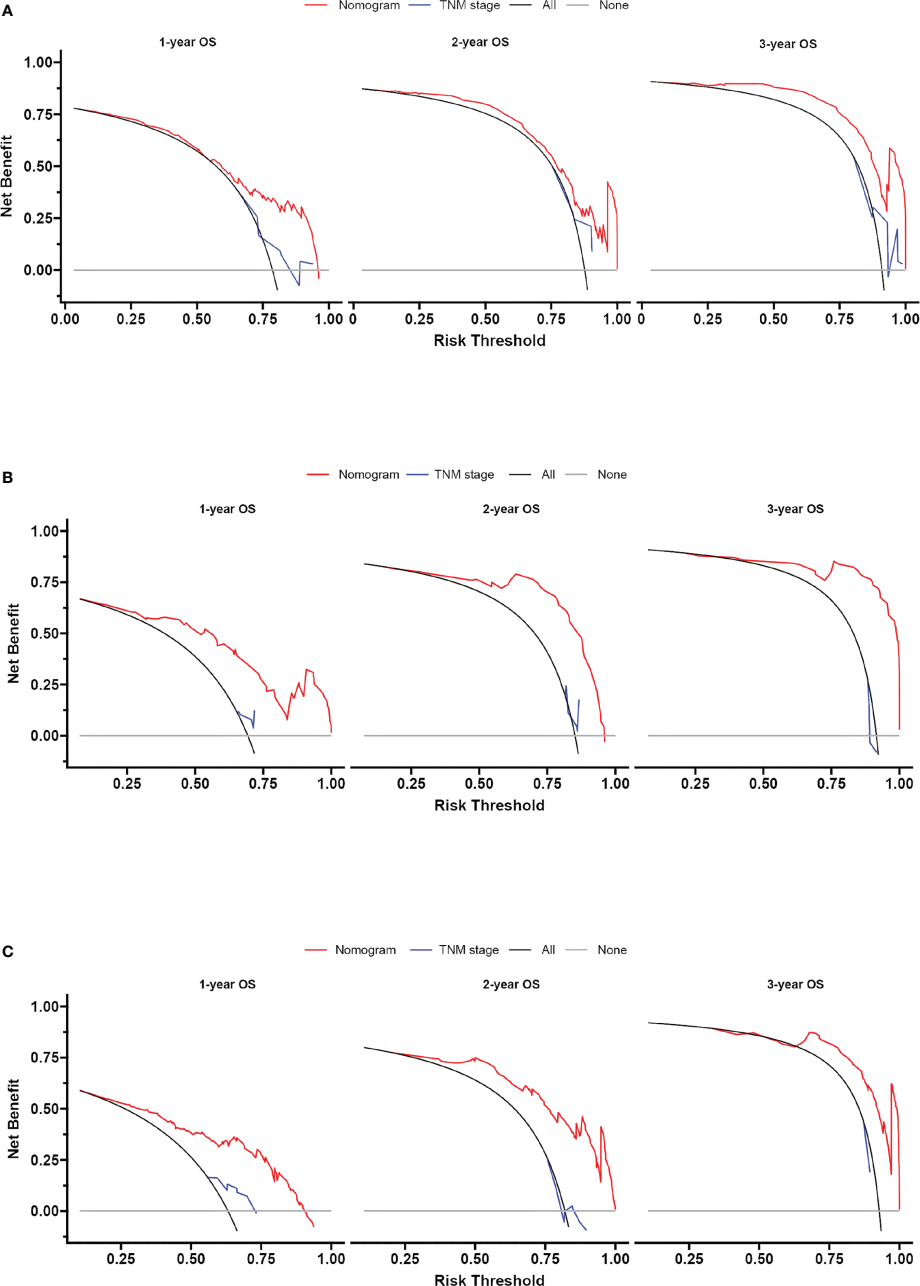
Decision curve analysis of the nomogram and TNM stage for survival prediction of PCBM patients. **(A)** 1-, 2- and 3-year survival benefit in the training cohort. **(B)** 1-,2- and 3-year survival benefit in the internal validation cohort. **(C)** 1-, 2- and 3-year survival benefit in the external validation cohort.

**Figure 8:**
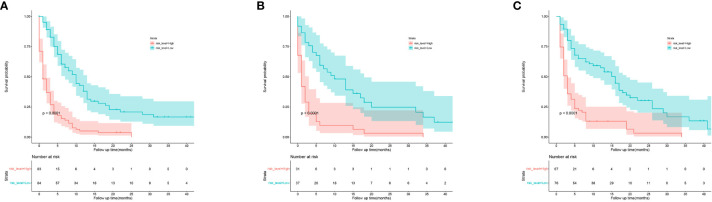
Kaplan-Meier curves of OS for patients in low-risk and high-risk groups. **(A)** the training cohort; **(B)** the internal validation cohort; **(C)** the external validation cohort.

Based on the independent predictors in the training cohort, we constructed a predictive nomogram model of PCBM (**Figure 4**). Similar to the previous web vision of diagnostic nomogram, the prognostic nomogram was made available *via* a free browser-based online calculator available at https://pcbm.shinyapps.io/PCBM_Cox_Nomo/. ROC analysis of the nomogram revealed that AUC of 1-, 2-and 3-year OS respectively reached 0.833, 0.888 and 0.874 in the training cohort (**Figures 5A**–**C**); 0.917, 0.905 and 0.992 in internal validation cohort (**Figures 5D**–**F**); and 0.909, 0.900 and 0.850 in external validation cohort (**Figures 5G**–**I**). As shown in **Figure 5**, area under the receiver operating characteristic curves of nomogram was obviously larger than that of TNM stage, suggesting that the nomogram has excellent accuracy. C-index and calibration curve were employed to verify the effectiveness of nomogram model training cohort. C-indices of training cohort, internal validation cohort, and external validation cohort were 0.795 (95%CI: 0.758-0.832), 0.800 (95%CI: 0.739-0.862), and 0.787 (0.746-0.828), respectively (**Table 5**). The calibration curve of nomogram revealed a strong consistency between actual observation and prediction (**Figure 6**). In addition, DCA was widely used to evaluate the clinical value of nomogram. As illustrated in **Figure 7**, the nomogram demonstrated a significant positive net benefit from the risk of death and is better than the traditional TNM staging system, indicating its great clinical practical value in predicting OS of PCBM. The Kaplan-Meier survival analysis of training cohort, internal validation cohort, and external validation cohort showed a distinct difference in survival rate between the three cohorts (**Figure 8**).

**Table 5:** The C-indices for predictions of overall survival.

	Training cohort	Validation cohort	External validation cohort
	HR (95%CI)	HR (95%CI)	HR (95%CI)
C-index	0.795 (0.758-0.832)	0.800 (0.739-0.862)	0.787 (0.746-0.828)

## Discussion

Skeletal metastases represent an underappreciated site of metastasis in patients with pancreatic cancer. Previous reports have estimated the prevalence to range from 5% to 20% (11, 12). Recently, there are many studies focused on treatment of PCBM, the researchers look forward to develop more individualized drug screening to replace traditional radiotherapy and chemotherapy (33, 34). PCBM is a relatively uncommon type of tumor bone metastasis. Due to its rarity, most studies on PCBM are case reports or single-institutional cohort studies (19–21). Therefore, it is critical to identify risk and prognostic factors of PCBM. To the best of our knowledge, our research is the first multicenter and comprehensive retrospective study to establish nomogram models to predict the risk and prognosis of BM in PC patients. Moreover, the ROC curve, calibration plot, and DCA revealed that the nomogram possesses considerable predictive power. This tool will make it easier to implement in clinical practice and enable doctors to determine the most appropriate treatment strategy for their patients.

As previously stated, there were few reports regarding the risk factors of PCBM. In a study of other digestive system tumors, the risk of developing BM was significantly associated with adenocarcinoma, gender, tumor size, poor grade, CEA positive, T1 stage, N1/N2 stage, brain metastasis, liver metastasis, and lung metastasis (29, 35, 36). In our study, risk factors for BM in PC patients included age, neuroendocrine carcinoma, primary site, N1 stage, radiotherapy, brain metastasis, liver metastasis and lung metastasis. As a result, clinicians should keep a keen sense and close attention to these risk factors for their PC patients. For these patients with potential risks, doctors should advise them to have PET-CT/ECT scans in a timely manner. According to our multivariate Cox analysis results, age, race, histological subtype, grade, surgery, chemotherapy, and lung metastasis were noteworthy predictors of BM in PC patients. Normal may appear to be a factor affecting prognosis, but in actual analysis, it is not. Based on the prognostic factors obtained in this study, doctors can more effectively evaluate the prognosis and provide clinical guidance for PC patients with BM.

In our study, younger PC patients had a higher risk of developing BM, but older ones with BM had a worse prognosis. Why might reducing age promote the development of metastasis? At the biological level some scientists would suggest two possible reasons, one related to the immune system and the other the mechanical properties of tissues (37). Recent study has shown that harnessing the immune system, such as CD4+T cells play an important role in metastatic process. The hypothesis suggests that age-related deterioration of the system may actually play a protective role by depriving the metastasis process of key immune-cellular components (38). Extra-cellular matrix (ECM) composition and remodeling are now considered necessary for tumorigenesis and progression of metastasis, including pre-metastatic niche construction. However, aging can also alter ECM through nonenzymatic glycosylation to reduce the activity of matrix modifying proteases, which are an essential factor in the selection of cancer cells during metastasis (39). Thus, age-dependent changes to ECM might also be protective. Several previous studies have also demonstrated that older age affects the prognosis of cancer patients with BM. In those studies, older patients indicated a low survival rate (40–42). We suspect that this phenomenon may be associated with low immunity and body degradation in elderly patients, but we cannot collect any further relevant information in the database.

In addition, compared with other people of color, black people with PCBM had the worst prognosis. Although the disparity in PC incidence and mortality between black and white patients in the United State (US) is narrowing, blacks continue to have a higher PC incidence and mortality rate than whites (43). In the multiracial environment of the US, cancer survival rates varied greatly among different races, and this difference was even more pronounced between whites and blacks (44, 45).

Adenocarcinoma was generally considered to be the most prevalent histological subtype of PC. To our knowledge, we reported for the first time the relationship between histological subtype and prognosis in PCBM patients. Adenocarcinoma alone was previously reported to exhibit the lowest median overall survival and poor prognosis in PC patients, which was extremely similar to our results (46). Concurrently, nomogram model suggested that grade was an independent prognostic risk factor for PCBM patients. At present, the first-line treatment of metastasis PC includes surgical resection and chemotherapy (47). Our study found that surgery and chemotherapy improved the survival prognosis of BM patients and further validated the reliability of this scheme in terms of clinical data.

Unlike BM, PC had a characteristic trend of preferential metastasis to the liver and lung, but brain metastasis of PC was almost as rare as BM (48–50). Interestingly, although brain and liver metastasis were risk factors for PCBM, they had no significant impact on the survival of PCBM patients. It was suggested that oncologists should take timely and effective measures to prevent metastasis in PC patients and pay attention to whether PC patients develop lung metastasis following BM.

In this study, we established a relatively complete evaluation system to accurately estimate the risk and prognosis of PCBM. We visualize these data using nomograms, which is more conducive to clinicians’ judgment and targeted treatment. To improve our model’s applicability, we used multicenter data from SEER database and validated the nomogram through internal and external validation. Due to the heterogeneity of data, we could not evaluate the nomogram only through internal validation; therefore, we utilized external validation to address this issue more effectively. External validation data were obtained from two large clinical hospitals in China to avoid selective bias. Surprisingly, nomogram showed satisfactory predictive value not only in training and internal validation cohort but also in external validation cohort.

Indeed, there are some limitations in our research. First of all, our external validation data comes from Asians, while the SEER database includes blacks, whites, and other people of color. Second, nomogram is based on retrospective studies, requiring further validation in prospective cohort and clinical trials. Third, we omitted certain potentially critical data, such as patient’s specific surgical procedure and chemotherapy regimen. Despite these limitations in this retrospective study, the nomogram model has practical utility in white, black and yellow population. The nomogram has been proved to be a efficient and instructive model, which can effectively assist clinicians in providing personalized treatment.

## Conclusion

To sum up, we first identified the risk factors of PCBM based on univariate and multivariate logistic regression analyses, and then determined prognostic factors using univariate and multivariate Cox regression analyses, resulting in the establishment of two nomograms. These nomograms can help clinicians effectively identify high-risk patients and treat them with different outcomes.

## Data Availability Statement

The datasets presented in this study can be found in online repositories. The names of the repository/repositories and accession number(s) can be found in the article/supplementary material.

## Ethics Statement

The studies involving human participants were reviewed and approved by the ethics committee of the Zhejiang Provincial People’s Hospital. The patients/participants provided their written informed consent to participate in this study. Written informed consent was obtained from the individual(s) for the publication of any potentially identifiable images or data included in this article.

## Author Contributions

QB conceived the idea, reviewed, and edited the manuscript. WZ wrote the manuscript. LJ, SZ, FF, and YZ contributed to literature retrieval. XW, JL, YT, and YK carried out research selection, data extraction, and statistical analysis. WZ and LJ prepared tables and figures. All authors contributed to this article and approved the submitted version.

## Funding

This research was supported in part by The Key Research and Development Program of Zhejiang Province under grant number 2021C03078, and the Natural Science Foundation of Zhejiang Province under grant number LQ19H160014, and Medical Health Science and Technology Project of Zhejiang Provincial Health Commission under grant number 2019KY320.

## Conflict of Interest

The authors declare that the research was conducted in the absence of any commercial or financial relationships that could be construed as a potential conflict of interest.

## Publisher’s Note

All claims expressed in this article are solely those of the authors and do not necessarily represent those of their affiliated organizations, or those of the publisher, the editors and the reviewers. Any product that may be evaluated in this article, or claim that may be made by its manufacturer, is not guaranteed or endorsed by the publisher.
